# Endometrial proteomic profile of patients with repeated implantation failure

**DOI:** 10.3389/fendo.2023.1144393

**Published:** 2023-07-31

**Authors:** Jing Yang, Linlin Wang, Jingwen Ma, Lianghui Diao, Jiao Chen, Yanxiang Cheng, Jing Yang, Longfei Li

**Affiliations:** ^1^ Reproductive Medical Center, Renmin Hospital of Wuhan University & Hubei Clinic Research Center for Assisted Reproductive Technology and Embryonic Development, Wuhan, China; ^2^ Department of Obstetrics and Gynecology, Renmin Hospital of Wuhan University, Wuhan, China; ^3^ Shenzhen Key Laboratory of Reproductive Immunology for Peri-implantation, Shenzhen Zhongshan Institute for Reproduction and Genetics, Shenzhen Zhongshan Urology Hospital, Shenzhen, China; ^4^ Guangdong Engineering Technology Research Center of Reproductive Immunology for Peri-implantation, Shenzhen Zhongshan Urology Hospital, Shenzhen, China; ^5^ Department of Reproductive Medicine, Chengdu XiNan Gynecological Hospital, Chengdu, China

**Keywords:** repeated implantation failure, endometrial receptivity, proteomic, TPPP3, HSD17B2

## Abstract

**Introduction:**

Successful embryo implantation, is the initiating step of pregnancy, relies on not only the high quality of the embryo but also the synergistic development of a healthy endometrium. Characterization and identification of biomarkers for the receptive endometrium is an effective method for increasing the probability of successful embryo implantation.

**Methods:**

Endometrial tissues from 22 women with a history of recurrent implantation failure (RIF) and 19 fertile controls were collected using biopsy catheters on 7-9 days after the peak of luteinizing hormone. Differentially expressed proteins (DEPs) were identified in six patients with RIF and six fertile controls using isobaric tag for relative and absolute quantitation (iTRAQ)-based proteomics analysis.

**Results:**

Two hundred and sixty-three DEPs, including proteins with multiple bioactivities, such as protein translation, mitochondrial function, oxidoreductase activity, fatty acid and amino acid metabolism, were identified from iTRAQ. Four potential biomarkers for receptive endometrium named tubulin polymerization-promoting protein family member 3 TPPP3, S100 Calcium Binding Protein A13 (S100A13), 17b-hydroxysteroid dehydrogenase 2 (HSD17B2), and alpha-2-glycoprotein 1, zinc binding (AZGP1) were further verified using ProteinSimple Wes and immunohistochemical staining in all included samples (n=22 for RIF and n=19 for controls). Of the four proteins, the protein levels of TPPP3 and HSD17B2 were significantly downregulated in the endometrium of patients with RIF.

**Discussion:**

Poor endometrial receptivity is considered the main reason for the decrease in pregnancy success rates in patients suffering from RIF. iTRAQ techniques based on isotope markers can identify and quantify low abundance proteomics, and may be suitable for identifying differentially expressed proteins in RIF. This study provides novel evidence that TPPP3 and HSD17B2 may be effective targets for the diagnosis and treatment of non-receptive endometrium and RIF.

## Introduction

Infertility is a common disease that troubles 10-15% couples of childbearing age globally (1, 2). In recent decades, the rapid development and application of assisted reproductive technology (ART) has provided the most effective methods for patients with infertility (3). However, there are still approximately 10% of infertile women undergoing IVF and suffering repeated implantation failure (RIF), termed women who are under the age of 40 but fail to achieve clinical pregnancy after transferring at least four high-quality embryos in at least three fresh or frozen cycles (4). Except for anatomical and chromosome abnormalities (such as uterine cavity abnormalities, hydrosalpinx and embryonic karyotype abnormalities), endometrial receptivity disorders are considered other contributor to RIF (5).

During the menstrual cycle, the endometrium undergoes periodic changes in menstrual repair, proliferation, and secretory differentiation under the control of continuous and complex timing interactions of female sex hormones (6). Endometrial receptivity refers to the maturation of the endometrium to support blastocyst acceptance and implantation, along with the endometrial epithelium and stromal cells, in a functional but transient state under the effects of ovarian estrogen and progesterone (7). This period is also termed the window of implantation (WOI), which usually occurs 6-8 days after the peak of luteinizing hormone (LH) and lasts about 3-5 days. To date, there are numerous molecular mediators reported to be involved in the regulation of endometrial receptivity, including cytokines, lipids, adhesion molecules, and growth factors (such as cyclooxygenase-2, Krueppel-like factor 5, leukemia inhibitory factor, interleukins, insulin-like growth factor-binding proteins, wingless/integrated factor, prolactin (PRL), vascular endothelial growth factor, and homeobox A10) (8–13). In pathological conditions such as chronic or acute inflammation, this window narrows or shifts to preclude normal implantation of the embryo, resulting in infertility or loss of pregnancy (7, 14). Approximately 18.6%-30.6% of patients undergoing *in vitro* fertilization and embryo transfer (IVF-ET) experience WOI translocation, resulting in impaired endometrial receptivity, considered one of the main causes of pregnancy failure when transferring high-quality embryos (15–19).

Examinations of endometrial receptivity by different methods, including endometrial biopsies and imaging examinations (such as ultrasound), have paved the way for treating unexplained RIF in the past few years. Although ultrasound is widely used and non-invasive, it is empirical and difficult to unify the evaluation criteria, with a poor ability to predict pregnancy rates and strong subjectivity (20). Since the 1920s, researchers have explored novel effective assessments of endometrial receptivity at the tissue level using endometrial microtubule structure (such as pinocytosis) and morphology (pinopodes), single molecule level by PRL, integrins, and multiple microarrays of -omics in recent years. In the past 20 years, microarrays (such as the endometrial receptivity array, ERA) and RNA sequencing techniques in whole-tissue transcriptional analysis have been widely used to assess endometrial receptivity and to determine the timing of IVF-ET (21). A recent study used single-cell RNA sequencing to analyze the presence of endometrial cells at WOI in patients with RIF and healthy controls and provided detailed molecular and cellular patterns of healthy and RIF endometrium at WOI (22). However, it is doubtful that techniques such as transcriptome-based ERA detection can improve the success rate of implantation (23).

Proteins in the endometrium are considered the most direct functional molecule of an organism and the final effector of transcriptional gene translation. The change in functional proteins directly affects the exertion of individual biological functions. Several studies have focused on whether there are identifiable proteome counterparts for transcriptional features. To compare and quantify the total expression profiles of complex protein mixtures, previous studies have typically used two-dimensional differential in-gel electrophoresis(DIGE), Nanobore liquid chromatography-tandem mass spectrometry (LC-MS/MS), and MALDI-TOF-TOF techniques (24–26). Isobaric tag for relative and absolute quantitation (iTRAQ) is a widely used labeling quantitative proteome technology developed by Applied Biosystems Inc. (ABI) in 2004. It involves a set of molecules binding to N-terminal amino groups and free amino groups of lysine residues, thereby labeling the peptides and achieving the purpose of proteome comparison between different samples by labeling different source samples with reagents of different molecular weights (27, 28). However, most of the biomarkers screened by proteomics have not been further verified by traditional molecular biology methods. There is still a long way to go before applying these markers in clinical practice.

Therefore, we employ proteomics to identify the differentially expressed proteins that may trigger RIF. To compare proteins in multiple groups of samples qualitatively and quantitatively, we use iTRAQ labeling combined with LC-MS/MS techniques to screen differential WOI endometrial proteins and identify potential biomarkers in patients with RIF. In this study, ProteinSimple Wes and immunohistochemistry are used to verify the screened protein molecules, providing experimental data for the establishment of endometrial receptivity indicators and the diagnosis of RIF. Potent protein biomarkers are further confirmed in decidual and endometrial tissues from other published databases.

## 2 Materials and methods

### 2.1 Study population and study design

This study was evaluated and approved by the ethics committee of Shenzhen Zhongshan Urology Hospital (Approval number: SZZSECHU-20180023). The study included 41 females who visited the Fertility Center of Shenzhen Zhongshan Urology Hospital from January 2018 to December 2019. These patients included 22 females with a history of RIF who experienced failed pregnancy in the next period after endometrial scratch and 19 fertile females who retrieved successful pregnancy in the next period after endometrial scratch. Herein, RIF was defined as women who experienced two or more retrieval cycles and transferred more than ten high-quality embryos without pregnancy (29). Among these patients, endometria from six RIF patients and six controls were subjected to proteomics analysis using isobaric tags for relative and absolute quantification (iTRAQ). All included patients were recruited for the validation of key deferentially expressed proteins (DEPs). **Table 1** presents the baseline characteristics of the study population.

**Table 1:** Baseline characteristics of the study population.

1a. Population for iTRAQ analysis			
Group	Normal pregnancy (n = 6)	Repeated implantation failure (n = 6)	*p*-value
Age (years)	33.2 ± 3.8	34.7 ± 5.8	0.61
BMI (kg/m2)	21.7 ± 2.0	20.2 ± 2.5	0.27
E2 (pg/mL)	30.0 ± 17.4	38.1 ± 20.0	0.47
P (ng/mL)	0.7 ± 1.1	0.4 ± 0.2	0.58
FSH (IU/L)	6.3 ± 1.9	8.4 ± 2.8	0.15
AMH (ng/mL)	2.8 ± 0.9	1.9 ± 0.7	0.14
LH (IU/L)	6.5 ± 4.2	3.8 ± 1.8	0.17
PRL (ng/mL)	18.8 ± 4.2	15.8 ± 7.8	0.47
T (ng/mL)	0.2 ± 0.1	0.2 ± 0.2	0.47
Endometrial thickness (mm)	9.8 ± 0.8	9.2 ± 1.4	0.34
1b. Population for validation			
Group	Normal pregnancy (n = 19)	Repeated implantation failure (n = 22)	*p*-value
Age (years)	33.2 ± 4.1	33.5 ± 4.2	0.82
BMI (kg/m2)	21.4 ± 2.3	21.2 ± 2.3	0.81
E2 (pg/mL)	39.0 ± 39.6	43.5 ± 41.8	0.74
P (ng/mL)	0.4 ± 0.6	0.5 ± 0.3	0.83
FSH (IU/L)	5.5 ± 1.7	6.6 ± 1.5	0.05
AMH (ng/mL)	2.8 ± 1.3	2.3 ± 1.3	0.30
LH (IU/L)	4.0 ± 3.2	4.0 ± 1.6	0.94
PRL (ng/mL)	16.6 ± 7.0	17.4 ± 7.5	0.77
T (ng/mL)	0.3 ± 0.2	0.3 ± 0.2	0.98
Endometrial thickness (mm)	10.3 ± 2.1	9.7 ± 2.0	0.31

### 2.2 Endometrial preparation program and sample collection

Natural cycles were used for the included patients before collecting the endometrium. During the cycles, progesterone (Dydrogesterone Tablet, Solvay Pharmaceuticals, Netherlands) was commenced at a dose of 20 mg per day for 18 days of the cycle and continued until the mid-luteal phase of the menstrual cycle. The endometrial tissues were collected by biopsy catheter (Gynetics, Lommel, Belgium) after 7-9 days of LH surge (LH 7-9) of the natural menstrual cycle before controlled ovarian hyperstimulation, which is confirmed by ultrasound monitoring of ovulation (30). Endometrial tissues were divided into two parts. One part was frozen at -80°C for iTRAQ or ProteinSimple Wes analysis. The other part was fixed in 4% formaldehyde for immunohistochemical (IHC) staining.

### 2.3 Measurements of serum hormone levels

On day 3 of the menstrual cycle, the concentrations of estradiol (E2), progesterone (P), follicle-stimulating hormone (FSH), anti-muellerian hormone (AMH), luteinizing hormone (LH), prolactin (PRL), and testosterone (T) in the sera of the patients were measured with Elecsys Estradiol III, progesterone II, FSH, AMH, LH, Prolactin II, and Testosterone II kit (Elecsys, Olathe, USA) and analyzed in an immunology analyzer (Roche Diagnostics, Basel, Switzerland).

### 2.4 iTRAQ-based proteomics analysis

Protein sample preparation, iTRAQ labeling, and liquid chromatography-tandem mass spectrometry (LC-MS/MS)-based protein identification were accomplished by FitGene Biotechnology Company (Guangzhou, China). Tissues were briefly lysed and sonicated for protein collection. Protein concentration was determined using the Bradford method. 8-plex iTRAQ labeling was performed following the manufacturer’s protocol (AB Scienx). Labeled peptides were fractionated, followed by LC-MS/MS analysis.

### 2.5 Biological analysis

iTRAQ experiments generate large datasets. Initially, MS-based proteomics was used to identify and quantify the proteins present in each sample. The original data from MS-based proteomics were analyzed using the ProteinPilot software to obtain reliable proteins. The analysis process was described as follows: DEPs screening was conducted based on the following inclusion criteria: (1) After the retrieval was completed, examining the Unused value for the original retrieval results, and setting Unused ≥ 1.3, so that the reliability of the protein was above 95%. (2) Removing the records beginning with “RRRR” and the reverse database from the search results. ProteinPilot™ performed false discovery rate (FDR) analysis to evaluate the accuracy of the retrieval results. The FDR analysis was realized by reverse library retrieval. Subsequently, DEPs were selected based on the following inclusion criteria: (1) coefficient of variation<0.5; (2) p-value<0.05 (t-test); (3) fold change ≥ 1.2 or ≤ 0.83. The detailed values (unused, citable accession, gene name, fold change, and p-value) for DEPs, including 15 proteins (fold change > 1.5 or < 0.67) are listed in **Supplementary Table 2**. The R package *pheatmap*, a free online platform (http://www.bioinformatics.com.cn), was used to visualize the DEPs, shown as volcano and heat maps (31).

Gene Ontology (GO) and Kyoto Encyclopedia of Genes and Genomes (KEGG) enrichment analyses were performed based on the DEPs. The bubble charts of GO terms were plotted by an online platform (http://www.bioinformatics.com.cn), whereas the chord diagrams of KEGG pathways were visualized by the *clusterProfiler* package. Furthermore, the protein-protein interaction (PPI) networks predicted by STRING (https://string-db.org/cgi/input.pl) were further visualized in Cytoscape software (version 3.8.2). The top ten hub proteins were identified from the PPI network of proteins through the calculation of degrees algorithm, and their expression levels were revealed in a heat map (32).

**Figure 1:**
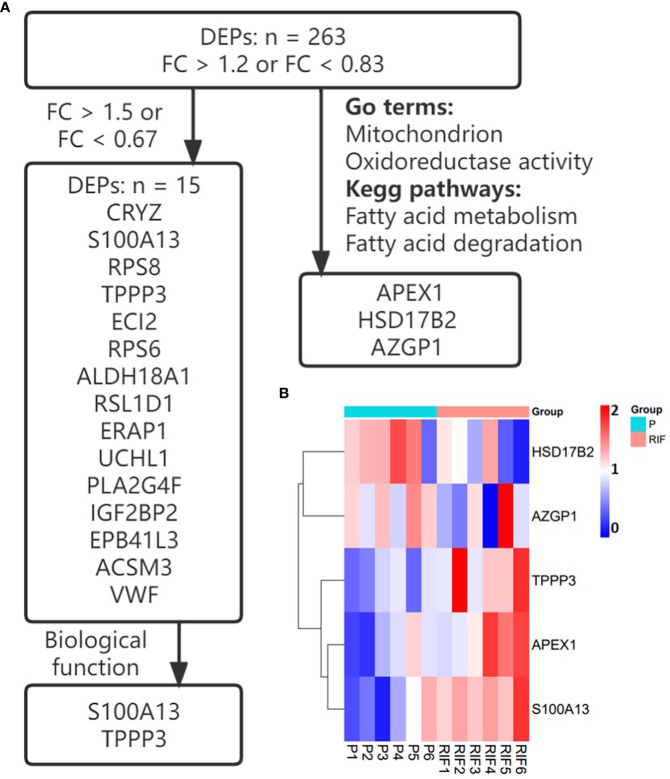
Selection and the expression of key proteins. **(A)** The screening process of key proteins. **(B)** Heatmap of the key proteins in patients with RIF compared with the normal pregnancy women.

Key proteins were further investigated according to fold change, biological function, and top enriched GO terms and KEGG pathways. The screening process is shown in **Figure 1A**, and the expression levels of key proteins are shown in a heat map. Key proteins including TPPP3 and HSD17B2 were further reviewed in published literature and the Human Protein Atlas (https://www.proteinatlas.org/). The expression levels of TPPP3 and HSD17B2 in decidual tissues were obtained from the research by Yu et al. (33). The immunoreactive pattern of TPPP3 and HSD17B2 in the endometrium were further contrasted with immunohistochemistry images for those antigens in endometrial sections available in the Human Protein Atlas database.

### 2.6 Immunohistochemistry

Specifically, the samples were fixed and embedded in paraffin for IHC staining. Using Bond polymer refine detection kit (Leica Microsystems, Germany), the samples were stained in an automatic immunostaining machine (Leica Bond Rx system, Germany). The images were collected by the multispectral panoramic tissue program analysis system Panel Detection 1.0 (Panovue, Beijing, China) and visualized by the HALO digital pathological image analysis platform (lndica Labs, USA). Detailed information regarding the primary antibodies used is provided in **Supplementary Table 1**.

### 2.7 ProteinSimple Wes analysis

Endometrial tissues were lysed with Radio Immunoprecipitation Assay (RIPA) Lysis Buffer (Beyotime, Shanghai, China), and the lysates were centrifuged 12000 × *g* for 15 min at 4°C to collect the supernatant. The BCA assay kit (Beyotime, Shanghai, China), was used to quantify the concentrations of total proteins. An automated Wes Capillary System (Protein Simple, San Jose, CA, 12-230 kDa kit cat. SM-W004) was used to detect the protein levels. Total protein loading was at a concentration of 1 μg/μL for each sample. Immunoblot analysis was executed using the Compass for Simple Western Program (ProteinSimple Wes, USA). More detailed information about primary antibodies is presented in **Supplementary Table 1**.

### 2.8 Statistical analysis

Statistical analyses were performed using SPSS for Windows (version 25.0, SPSS Inc., Chicago, IL). Variables were analyzed by student’s t-test, and shown as mean ± standard deviation. In all comparisons, a two-tailed *p*-value < 0.05 was considered statistically significant.

## 3 Results

### 3.1 Baseline characteristics of the study population

Baseline characteristics, including maternal age, body mass index (BMI), baseline serum hormone levels (E2, P, FSH, AMH, LH, PRL and T), and endometrial thickness were comparable between the RIF group and the fertile controls (**Table 1**, *p* ≥ 0.05 for each parameter). **Table 1** presents the baseline characteristics of the study population.

### 3.2 Functional enrichment analysis and protein-protein interaction network analysis of DEPs

**Figure 2:**
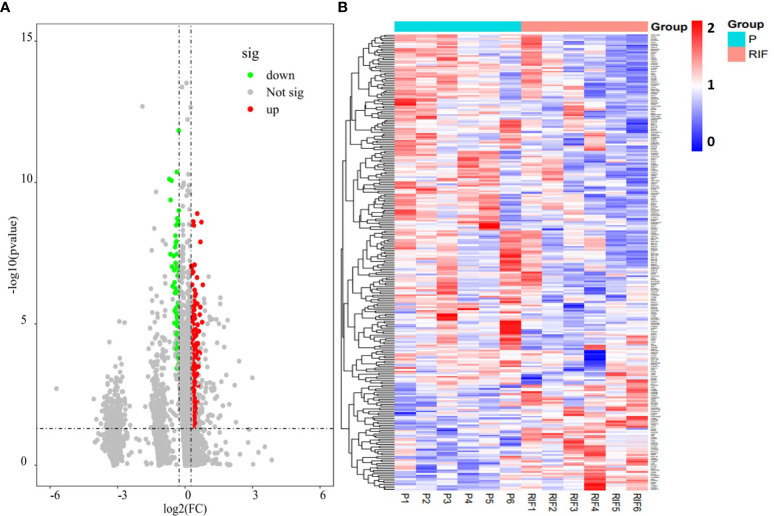
Endometrial proteomic profile of the study population. **(A)** Volcano plot of all endometrium-expressed proteins (P divided by RIF, log2-fold-change threshold = 1). **(B)** Heatmap of the DEPs identified in patients with RIF compared with the normal pregnancy women.

**Figure 3:**
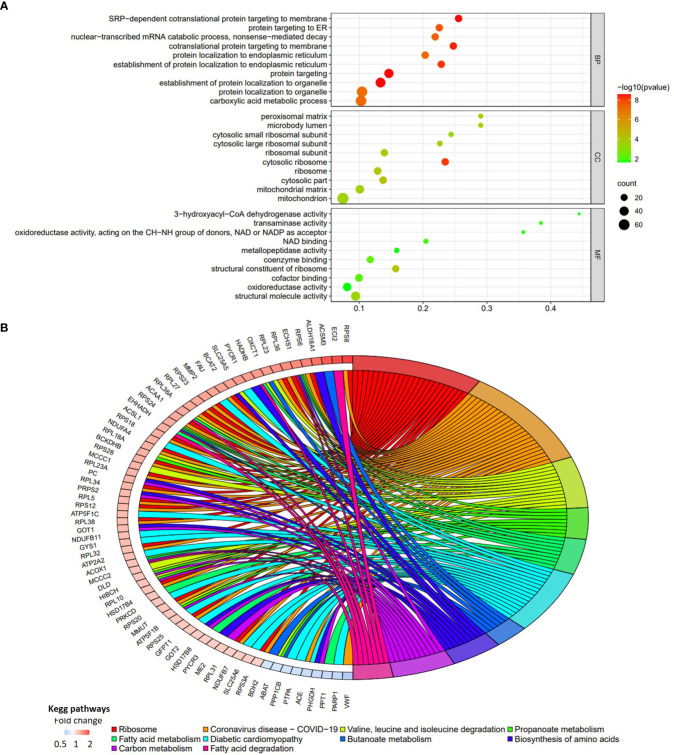
Functional enrichment analyses of the DEPs in patients with RIF compared with the normal pregnancy females. **(A)** The bubble chart of top 10 GO terms for biological process, cellular component, and molecular function. **(B)** The chord diagram of top 10 KEGG pathways (P vs. RIF). Fold change is calculated by dividing the expression of DEP in control group by the corresponding value in RIF group.

**Figure 4:**
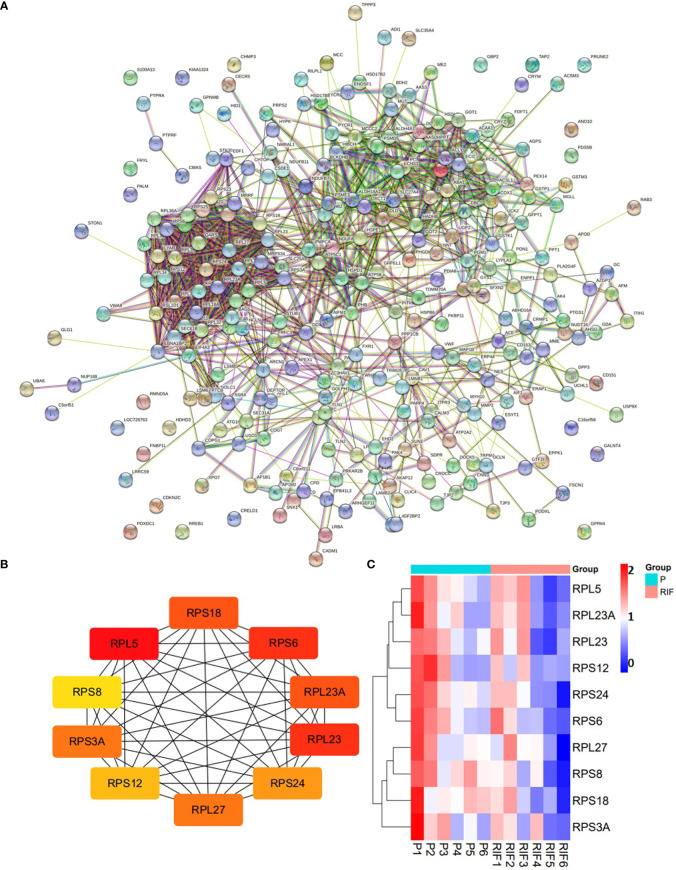
Protein-protein interaction network of the DEPs **(A)**, and top 10 hub proteins among the DEPs **(B)**. **(A)** medium confidence (0.400). **(C)** Heatmap of the 10 hub proteins identified in RIF patients compared with the normal pregnancy females.

Totally, there were 263 DEPs identified in the endometrial tissues of RIF patients compared with those from fertile controls, including 202 down-regulated proteins and 61 up-regulated proteins (**Figure 2A**; **Supplementary Table 2**). These DEPs were expressed at exactly opposite levels between RIF and control group (**Figure 2B**). GO term enrichment showed that DEPs were primarily enriched in translation, ribosomal subunits, carboxylic acid metabolic processes, oxidoreductase activity, and mitochondrial function (**Figure 3A**). KEGG pathways were mainly enriched in ribosome, immune response, and metabolism, including fatty acid, amino acid, carbon, and other organic acid metabolism. (**Figure 3B**). The top ten hub proteins were ribosomal proteins, including RPL5, RPL23A, RPL23, RPL27, RPS3A, RPS6, RPS8, RPS12, RPS18 and RPS24 (**Figure 4**). These hub proteins were all down-regulated in the endometria of patients with RIF, suggesting that deficiency of ribosomal protein small subunit (RPS) and ribosomal protein large subunit (RPL) may be associated with impaired endometrial receptivity.

### 3.3 Validation of key proteins

**Figure 5:**
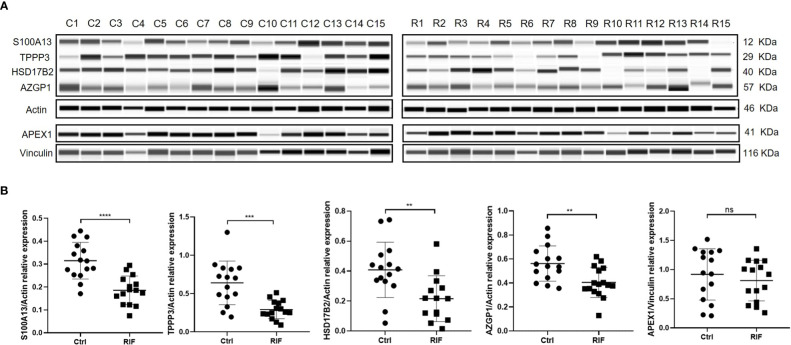
ProteinSimple Wes imaging **(A)** and quantitative analysis **(B)** of the expression of S100A13, TPPP3, HSD17B2, AZGP1 and APEX1 in endometrium of the study population. n = 15 per group. ns, not significant, ** p<0.01, *** p < 0.001, **** p < 0.0001.

**Figure 6:**
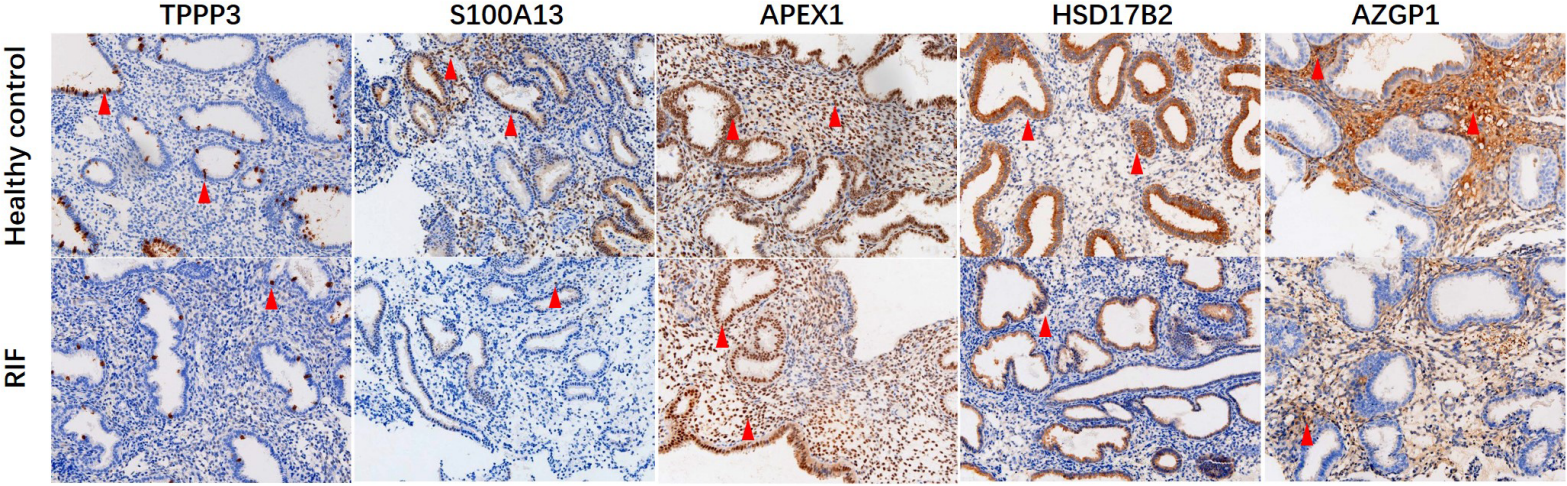
Immunohistochemical staining of TPPP3, S100A13, APEX1, HSD17B2 and AZGP1 in endometrial tissues. Red arrows indicate positive cells. Magnification, × 200.

**Figure 7:**
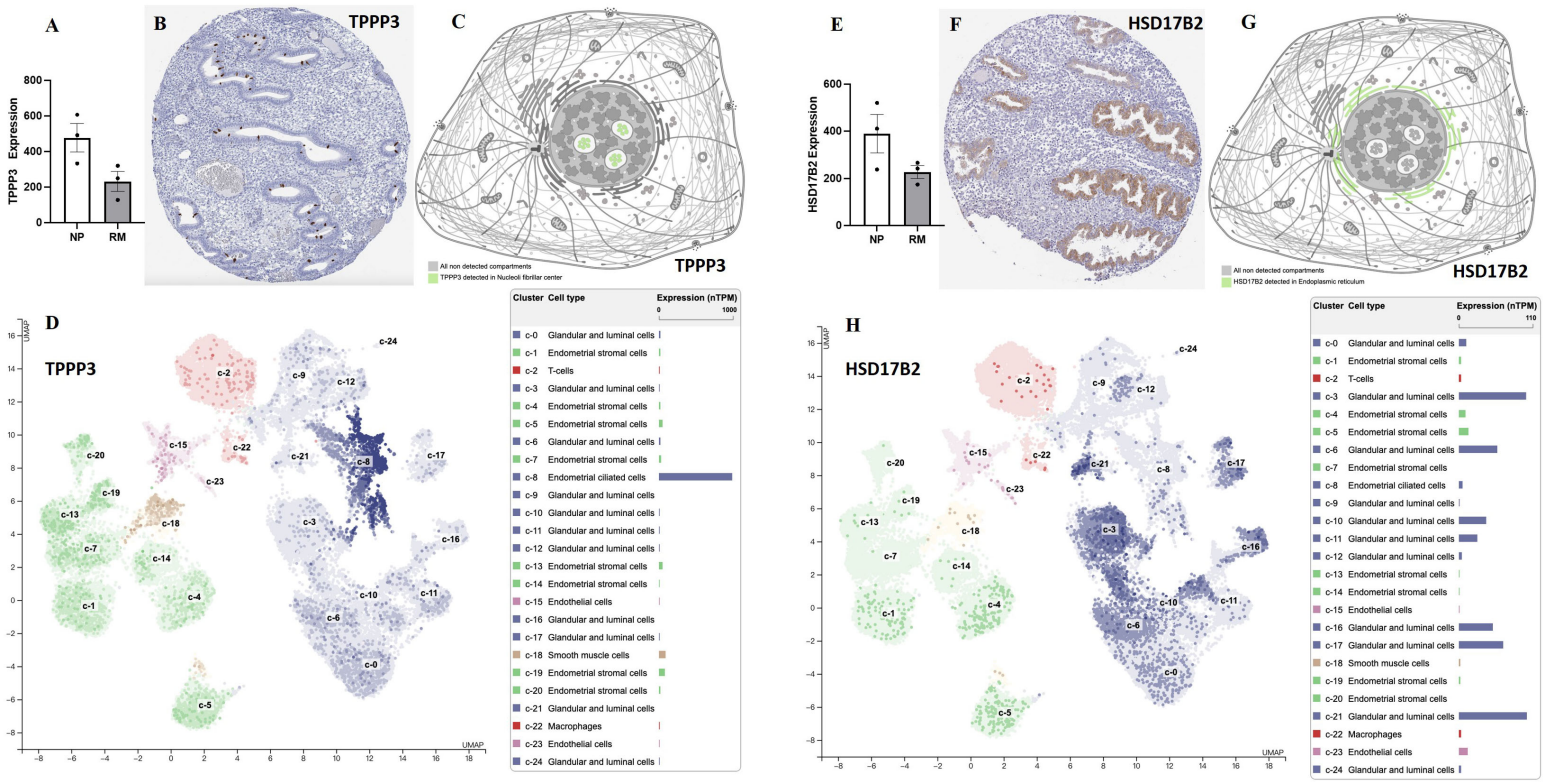
The expression levels of TPPP3 and HSD17B2 in decidual and endometrial tissues. **(A)** The expression levels of TPPP3 in decidual tissues of females with normal pregnancy or recurrent miscarriage from Yu et al., (33). **(B)** The expression of TPPP3 in endometrium. **(C)** Subcellular localization of TPPP3. **(D)** The expression levels of TPPP3 in endometrial cells (single cell-RNA seq). **(E)** The expression levels of HSD17B2 in decidual tissues of females with normal pregnancy or recurrent miscarriage from Yu et al., (33). **(F)** The expression of HSD17B2 in endometrium. **(G)** Subcellular localization of HSD17B2. **(H)** The expression levels of HSD17B2 in endometrial cells (single cell-RNA seq). Data in **(B–D, F–H)** were gained from database of Human Protein Atlas.

Key proteins were further screened according to fold change (> 1.5 or < 0.67), biological functions (cell proliferation or apoptosis, immune response, or angiogenesis), and top-enriched GO terms and KEGG pathways. The screening process is shown in **Figure 1A**. iTRAQ analysis showed that the expressions of HSD17B2 and AZGP1 decreased in the endometria from RIF patients compared with those from fertile controls, whereas TPPP3, APEX1, and S100A13 increased in the RIF group (**Figure 1B**). Both ProteinSimple Wes analysis and IHC staining verified the down-regulation of HSD17B2, AZGP1, TPPP3, and S100A13 in the endometrial tissues of the RIF patients compared to those from the fertile controls (**Figures 5A, B**, **6**). Interestingly, the expression levels of TPPP3 and HSD17B2 also decreased in the decidual tissues of patients with miscarriage (**Figures 7A, E**). These two proteins were further examined in the Human Protein Atlas. The signals of TPPP3 were primarily located in the cell nucleus and expressed in the endometrial ciliated cells (**Figures 7B–D**). The signals of HSD17B2 were mostly located in endoplasmic reticulum and expressed in glandular and luminal cells (**Figures 7F–H**).

## Discussion

Although ART has made rapid progress in the past 40 years, the clinical pregnancy rate and live birth rate of ART remain at only 54.7% and 45.0%, respectively (34). RIF and repeated pregnancy failure remain the common reproductive disorders that significantly affect the physical and mental health of 10-15% of couples of childbearing age (35, 36). Poor endometrial receptivity is considered the main reason for the decrease in pregnancy success rates in patients suffering from RIF. Therefore, exploring the functional protein molecular changes of endometrium during WOI is crucial for the identification of biomarkers for endometrial receptivity and the diagnosis and treatment of pathogenic RIF. Therefore, we employed iTRAQ to compare the proteomics of endometrial receptive states in the endometria from fertile controls and RIF patients. Compared with the endometria from normal pregnant people, there are 263 DEPs in endometrial tissues of patients with RIF, which are mainly enriched in ribosomal subunits and metabolic process. Furthermore, the expression patterns of AZGP, HSD17B2, S100A13, and TPPP3 in RIF patients were further verified by Protein Simple Wes analysis and IHC staining as well as in other published databases.

Recently, researchers sought to evaluating whole genome and transcriptome through ERA or RNA-seq in the endometria from RIF to identify the abnormal interruptions in endometrial receptivity gene expression profiles (17, 19, 37–39). However, the correlation between mRNA and the translated protein derivatives is usually very low. The transcriptional results cannot echo for the protein changes caused by post-transcriptional and post-translational regulations, especially in the endometrium (40, 41). Therefore, proteomics might provide more physiological information than genomics. iTRAQ techniques based on isotope markers can identify and quantify low abundance proteomics, and may be suitable for identifying differentially expressed proteins in menstrual endometrium (42, 43).

In this study, iTRAQ LC-MS/MS was employed to screen specific proteins related to endometrial receptivity. We detected more than 5000 proteins in each iTRAQ experiment, and screened 263 DEPs after three parallel experiments. Among all the DEPs identified in this study, the central protein of the protein interaction network is ribosomal proteins, including RPL and RPS. Our data showed that both RPL and RPS are down-regulated in the endometria from patients with RIF, suggesting that defects in ribosomal subunits may lead to failed embryo implantation by impairing endometrial receptivity. Simultaneously, DEPs were primarily enriched in the translation, ribosomal subunits and synthesis of GO biology, and the results from KEGG pathway analysis also echo this observation. Moreover, GO molecular functional association network and KEGG pathway analysis indicated that DEPs were primarily rich in carboxylic acid metabolism, oxidoreductase activity and mitochondrial function, and active in immune response and metabolic pathways, including fatty acid, amino acid, carbon metabolism, and other organic acid metabolism. This suggests that the mechanism of altering cell metabolism may be closely related to the disturbance of endometrial receptivity. Material metabolism and mitochondrial function are very important for embryo implantation and pregnancy. Studies have shown that the samples from patients with RIF exhibit obvious mitochondrial dysfunction characteristics, and biological metabolic processes, such as hormones and lipids, also differ significantly from samples from patients with normal pregnancy (44, 45). Several recent studies have provided additional evidences for this observation. Mediators of glycose and lipid metabolism (such as lysophosphatidic acid receptor 3 and glucose transporter 1) and cholesterol-derived steroids (including progesterone and estrogen) were linked with endometrial receptivity, embryonic septum, and decidualization. These mediators regulated the organization and function of the endometrium during preparation for blastocyst implantation (8, 46–48).

The key functional proteins according to the multiple changes were further enriched in GO terms and KEGG pathway. ITRAQ analysis showed that compared with the fertile control, the protein levels of HSD17B2 and AZGP1 in the endometria from RIF were down-regulated, whereas the expression of TPPP3, APEX1, and S100A13 were up-regulated. These proteins are primarily involved in biological processes such as cell proliferation and apoptosis, immune response or angiogenesis, which play important roles in the process of embryo implantation. Consequently, we expanded the sample size and further verified the expression of these five key proteins by Protein Simple Wes and IHC staining. The results showed that the protein levels of HSD17B2, AZGP1, TPPP3, and S100A13 in endometrium of RIF patients were significantly lower than patients with normal pregnancy.

TPPP3 is a member of the tubulin polymerization promoting protein family, which can bind to tubulin, promote microtubule aggregation, and maintain the stability and integrity of the microtubule system (49). Microtubule is the main component of mitotic spindle, which controls cell division and chromosome segregation (50). Studies have revealed that TPPP3 gene can inhibit cell proliferation, induce apoptosis and cell cycle arrest, and inhibit tumor growth (51–53). TPPP3 protein is also involved in the development and regeneration of musculoskeletal and nervous system (54, 55). Additionally, TPPP3 participates in palmitic acid metabolism to activate oxidative stress and induce cell damage (56). Recent studies found that inhibition of *TPPP3* expression weakens β-catenin/NF- kappa B/COX-2 signal transduction and damage decidualization in endometrial stromal cells, and *TPPP3* knockout can lead to embryo implantation failure and inhibit the expression of receptive markers (57, 58). This is consistent with our verification results that the expression of TPPP3 protein is significantly down-regulated in RIF patients. These results suggest that TPPP3 plays an essential role in embryo implantation and maintenance of pregnancy.

S100A13 is a small calcium (Ca^2+^)-binding protein belonging to the S100 family. S100 proteins play fundamental roles in a series of cellular processes, such as calcium homeostasis, cell proliferation, apoptosis, and inflammation (59). The abnormal regulation of S100 proteins, including S100A13, is related to tumor cell proliferation, immune infiltration, and change of chemosensitivity (60–62). Transcriptome or proteomics found that *S100P*, *S100A4*, *S100A6*, *S100A8*, *S100A9*, *S100A10*, *S100A11*, *S100A13*, and *S100A16* were significantly down-regulated in endometria of RIF or failed pregnancy, which are predictors of poor endometrial receptivity (22, 25, 26, 42, 63–65). Additionally, S100A10 could affect the decidualization and secretory transformation of primary endometrial stromal cells and epithelial cells, and coordinate Annexin A2 to promote embryo implantation (25, 66). *S100A11* knockout in the uterus hindered mouse embryo implantation and adversely affected the expression of endometrial receptivity-related factors and the immune response of human endometrial cells (67). This study showed that *S100A13* was significantly down-regulated in RIF patients, which was consistent with the above reports, suggesting that S100A13 and its family members may be major contributors in the establishment of endometrial receptivity.

HSD17B is an enzyme responsible for the synthesis and inactivation of estrogen and androgen. In addition to producing active progesterone, HSD17B2 oxidizes estradiol to estrone and testosterone and androstenediol to androstenedione dehydroepiandrosterone (68–70). It plays an important role in sex hormone-related diseases such as breast cancer, endometrial cancer and osteoporosis (69, 71–73). In normal conditions, progesterone acts on endometrial stromal cells and induces the secretion of paracrine factors, and afterward stimulates adjacent epithelial cells to express enzyme HSD17B2, which rapidly metabolizes biologically potent estrogen E2 into weak estrogen. However, in pathological condition such as endometriosis, progesterone is unable to induce the expression of *HSD17B2* in epithelial cells owing to the defect of stromal cells. The metabolism of E2 in endometriosis is insufficient, resulting in a high local concentration (74–76). Our data showed that HSD17B2 in endometrial tissues from RIF patients was significantly lower than those in normal pregnant women. However, serum estradiol levels in RIF patients were slightly higher than fertile controls without any significance. This indicates that the abnormal decrease of *HSD17B2* is the possible cause of poor endometrial receptivity.

AZGP1, also known as zinc- α 2-glycoprotein (ZAG), is a secretory adipose factor regulated by thyroid hormones, androgens, and glucocorticoids (77). The change of AZGP1 level is closely associated with obesity and related complications, such as diabetes, obesity and polycystic ovary syndrome (78, 79). AZGP1 participates in metabolic processes such as lipolysis and glucose transport, and acts as a tumor suppressor in malignant tumors such as pancreatic ductal adenocarcinoma and hepatocellular carcinoma (77, 78). The structure and folding of ZAG are similar to those of MHC-I antigen presentation molecules. Decrease of *AZGP1* expression is accompanied with a significant increase of inflammatory factors. Collectively, AZGP1 may play an important role in the regulation of local immune responses (77, 80). Based on previous research, we hypothesize that poor endometrial receptivity in patients with RIF may be associated with a persistent hyperinflammatory endometrial environment and metabolic abnormalities mediated by downregulation of the AZGP1 protein.

We re-analyzed the data from a recurrent abortion DNA methylome and transcriptome from Yu et al., (33) and discovered that the expression levels of TPPP3 and HSD17B2 significantly decreased in decidual tissues of patients with recurrent abortion. These observations suggest that TPPP3 and HSP70B2 might play an important role in embryo implantation and pregnancy maintenance. Furthermore, we reviewed the expression patterns of these two proteins in the public database (Human Protein Atlas). TPPP3 is primarily located in the nucleus and expressed in endometrial ciliated cells, whereas HSD17B2 is primarily located in the endoplasmic reticulum and abundantly expressed in glandular and luminal cells. The expression pattern is consistent with our observations, indicating that our verification results are reliable and credible.

Although this study may provide new targets for the diagnosis and treatment of RIF, it has some limitations. First, the sample size of this study is inadequate, and some unknown bias may exist. This may also be the main reason for the inconsistent expression trend of two key proteins, TPPP3 and S100A13, in specimen verification and proteomics analysis. To avoid the deviation caused by individual heterogeneity, large sample sizes may be required to further verify the findings. Second, proteomic specimens are obtained by invasive endometrial biopsy, whether DEPs including TPPP3 and HSD17B2 are useful as an important reference for endometrial receptivity in non-invasive acquisition of samples of blood, urine and cervical swabs needed to be verified in the coming study. Finally, the molecular mechanism and treatment strategies of the key proteins including TPPP3 and HSD17B2 should be further verified in animal studies.

In summary, our results suggest that endometrial TPPP3, S100A13, HSD17B2, and AZGP1 levels may be key biochemical markers for endometrial receptivity and the diagnosis of RIF. Our results provide novel experimental evidences for the establishment of endometrial receptivity indicators and a new outlook for the pathogenesis of poor endometrial receptivity in patients with RIF.

## Data availability statement

The datasets presented in this study can be found in online repositories. The names of the repository/repositories and accession number(s) can be found below: https://ngdc.cncb.ac.cn, PRJCA014430.

## Ethics statement

The studies involving human participants were reviewed and approved by the ethics committee of Shenzhen Zhongshan Urology Hospital (Approval number: SZZSECHU-20180023). The patients/participants provided their written informed consent to participate in this study.

## Author contributions

JY (1st author), LW, and LL conceived the original idea and the structure of the manuscript. LW and JM performed the statistical analysis. JY (1st author) and LW drafted the first version of the manuscript. JY (1st author), LW, and JM developed the figures. LD and JC provided critical feedback and helped shape the manuscript. LL, JY (7th author), and YC supervised and revised the manuscript. All authors contributed to the article and approved the submitted version.
