# Supplementary material for: Endometrial proteomic profile of patients with repeated implantation failure
Source: Front Endocrinol (Lausanne). 2023 Jul 31;14:1144393. doi: 10.3389/fendo.2023.1144393 (PMC10424929; doi:10.3389/fendo.2023.1144393)
Supplement: Supplementary file 1 [file Table_1.docx]

Supplementary Material

Endometrial proteomic profile of patients with repeated implantation failure

**Jing Yang^a,1,†^, Linlin Wang^2,3,4, †^, Jingwen Ma^5, †^, Lianghui Diao^3,4^, Jiao Chen^1^, Yanxiang Cheng^2,^*, Jing Yang^b,1,^*, Longfei Li^3,4,^***

*** Correspondence:** Longfei Li: lilongfei2005@163.com;

Jing Yang: dryangjing@whu.edu.cn;

Yanxiang Cheng: yanxiangCheng@whu.edu.cn

# Supplementary Tables

**Supplementary Table 1. Summary of the primary antibodies and methods for validation**

| Antibody | Source | Catalog | Dilution (WB) | Dilution (IHC) | Antigen retrieval (IHC) |
| --- | --- | --- | --- | --- | --- |
| S100A13 | ThermoFisher | PA5-54033 | 50X | 1000X | Citrate (5.80-6.00) |
| TPPP3 | ThermoFisher | PA5-93213 | 50X | 800X | Citrate (5.80-6.00) |
| APEX1 | ThermoFisher | PA5-80342 | 50X | 2000X | Citrate (5.80-6.00) |
| HSD17B2 | Novus | NBP1-92011 | 20X | 100X | Citrate (5.80-6.00) |
| AZGP1 | Abcam | ab180574 | 50X | 1000X | Citrate (5.80-6.00) |
| ACTIN | Cell signaling technology | 3700S | 1000X | / | / |
| VINCULIN | Abcam | ab129002 | 1000X | / | / |

**Supplementary Table 2. Differentially expressed proteins detected in endometrium of RIF patients compared with normal pregnancy females**

| **Number** | **Unused** | **Citable Accession** | **Gene Name** | **Fold change (P:RIF)** | ***p*-value** |
| --- | --- | --- | --- | --- | --- |
| 1 | 20.74 | P62241 | RPS8 | 1.729 | 0.000000419 |
| 2 | 28.20 | O75521 | ECI2 | 1.694 | 0.000008720 |
| 3 | 8.34 | Q53FZ2 | ACSM3 | 1.656 | 0.000000003 |
| 4 | 40.01 | Q9NZ08 | ERAP1 | 1.606 | 0.000000013 |
| 5 | 28.87 | Q08257 | CRYZ | 1.603 | 0.000002561 |
| 6 | 42.41 | P54886 | ALDH18A1 | 1.580 | 0.000107732 |
| 7 | 19.56 | O76021 | RSL1D1 | 1.572 | 0.000004722 |
| 8 | 17.29 | Q9Y2J2 | EPB41L3 | 1.536 | 0.000018352 |
| 9 | 18.33 | P62753 | RPS6 | 1.517 | 0.000325087 |
| 10 | 15.18 | Q68DD2 | PLA2G4F | 1.504 | 0.000167916 |
| 11 | 9.67 | Q9NW15 | ANO10 | 1.497 | 0.001499848 |
| 12 | 4.26 | Q99685 | MGLL | 1.490 | 0.000018358 |
| 13 | 48.39 | P30084 | ECHS1 | 1.484 | 0.000048919 |
| 14 | 14.79 | P37059 | HSD17B2 | 1.478 | 0.000096464 |
| 15 | 8.30 | Q9NYL4 | FKBP11 | 1.477 | 0.000051883 |
| 16 | 8.93 | Q9Y3U8 | RPL36 | 1.472 | 0.000010745 |
| 17 | 21.01 | A3KMH1 | VWA8 | 1.459 | 0.000001932 |
| 18 | 36.48 | Q5JRX3 | PITRM1 | 1.456 | 0.000000001 |
| 19 | 17.23 | Q8TD43 | TRPM4 | 1.455 | 0.006007353 |
| 20 | 17.74 | P62829 | RPL23 | 1.448 | 0.000595034 |
| 21 | 11.69 | Q14894 | CRYM | 1.445 | 0.000000230 |
| 22 | 4.92 | Q8IV36 | HID1 | 1.417 | 0.000017906 |
| 23 | 32.68 | P55809 | OXCT1 | 1.411 | 0.006319341 |
| 24 | 47.68 | P55084 | HADHB | 1.408 | 0.000025443 |
| 25 | 7.81 | Q8TB45 | DEPTOR | 1.408 | 0.000049923 |
| 26 | 18.46 | P32322 | PYCR1 | 1.404 | 0.000645705 |
| 27 | 32.90 | P05141 | SLC25A5 | 1.403 | 0.000124349 |
| 28 | 44.31 | Q99623 | PHB2 | 1.388 | 0.000000875 |
| 29 | 2.59 | O15085 | ARHGEF11 | 1.386 | 0.000015641 |
| 30 | 33.28 | Q9UDY2 | TJP2 | 1.382 | 0.000624210 |
| 31 | 12.92 | O15382 | BCAT2 | 1.381 | 0.000069108 |
| 32 | 15.02 | P58107 | EPPK1 | 1.381 | 0.005969876 |
| 33 | 3.01 | P62861 | FAU | 1.381 | 0.000145279 |
| 34 | 9.93 | P08253 | MMP2 | 1.379 | 0.015144273 |
| 35 | 13.25 | P62266 | RPS23 | 1.377 | 0.000727994 |
| 36 | 5.68 | P55008 | AIF1 | 1.372 | 0.009738343 |
| 37 | 42.72 | Q9NY33 | DPP3 | 1.372 | 0.009595001 |
| 38 | 17.45 | Q9HBL8 | NMRAL1 | 1.370 | 0.000385602 |
| 39 | 18.36 | O95049 | TJP3 | 1.364 | 0.000068028 |
| 40 | 30.84 | P19827 | ITIH1 | 1.363 | 0.032512367 |
| 41 | 12.52 | P61353 | RPL27 | 1.359 | 0.001131601 |
| 42 | 55.22 | Q9Y678 | COPG1 | 1.356 | 0.000001029 |
| 43 | 6.00 | Q96NB2 | SFXN2 | 1.355 | 0.000302332 |
| 44 | 5.89 | P83881 | RPL36A | 1.354 | 0.006917760 |
| 45 | 25.29 | P09110 | ACAA1 | 1.353 | 0.000006375 |
| 46 | 9.53 | Q9Y3Y2 | CHTOP | 1.353 | 0.008446562 |
| 47 | 8.48 | P62847 | RPS24 | 1.352 | 0.000027778 |
| 48 | 36.40 | P02774 | GC | 1.350 | 0.011345887 |
| 49 | 29.50 | P02765 | AHSG | 1.346 | 0.012393303 |
| 50 | 11.45 | P27169 | PON1 | 1.343 | 0.000000082 |
| 51 | 8.51 | Q08426 | EHHADH | 1.343 | 0.000000637 |
| 52 | 89.91 | P10809 | HSPD1 | 1.341 | 0.004290694 |
| 53 | 12.61 | Q9H7D0 | DOCK5 | 1.339 | 0.000078826 |
| 54 | 3.05 | Q9Y6Q2 | STON1 | 1.338 | 0.000332569 |
| 55 | 33.42 | P33121 | ACSL1 | 1.336 | 0.000285514 |
| 56 | 20.95 | P62269 | RPS18 | 1.335 | 0.000004971 |
| 57 | 23.33 | Q9UKK3 | PARP4 | 1.335 | 0.031463567 |
| 58 | 48.45 | O60763 | USO1 | 1.334 | 0.000370473 |
| 59 | 25.96 | Q14978 | NOLC1 | 1.334 | 0.000001569 |
| 60 | 8.76 | O00483 | NDUFA4 | 1.333 | 0.021637186 |
| 61 | 15.37 | Q02543 | RPL18A | 1.333 | 0.018455082 |
| 62 | 7.23 | O94915 | FRYL | 1.333 | 0.004260634 |
| 63 | 48.12 | P48444 | ARCN1 | 1.327 | 0.001337068 |
| 64 | 19.87 | O95870 | ABHD16A | 1.327 | 0.000003191 |
| 65 | 8.12 | P21953 | BCKDHB | 1.326 | 0.000002361 |
| 66 | 8.99 | P62857 | RPS28 | 1.325 | 0.002244954 |
| 67 | 5.57 | Q5EBL4 | RILPL1 | 1.322 | 0.001054904 |
| 68 | 7.51 | Q9Y3E7 | CHMP3 | 1.321 | 0.000256729 |
| 69 | 27.41 | Q96RQ3 | MCCC1 | 1.317 | 0.000060173 |
| 70 | 33.16 | O75534 | CSDE1 | 1.317 | 0.026327456 |
| 71 | 16.38 | O75976 | CPD | 1.317 | 0.000025154 |
| 72 | 9.52 | P37268 | FDFT1 | 1.316 | 0.004167360 |
| 73 | 2.82 | P23508 | MCC | 1.316 | 0.000002314 |
| 74 | 18.80 | P62750 | RPL23A | 1.313 | 0.013619540 |
| 75 | 51.30 | P11498 | PC | 1.313 | 0.000000003 |
| 76 | 21.96 | Q5T0N5 | FNBP1L | 1.313 | 0.014322210 |
| 77 | 21.74 | P43652 | AFM | 1.311 | 0.033761441 |
| 78 | 23.82 | Q7Z4W1 | DCXR | 1.310 | 0.001001030 |
| 79 | 58.98 | Q15084 | PDIA6 | 1.310 | 0.001437682 |
| 80 | 9.32 | P49207 | RPL34 | 1.306 | 0.018325026 |
| 81 | 10.45 | O75608 | LYPLA1 | 1.306 | 0.002713543 |
| 82 | 29.13 | Q9BS26 | ERP44 | 1.305 | 0.046388402 |
| 83 | 11.92 | Q9H993 | ARMT1 | 1.303 | 0.000907499 |
| 84 | 25.30 | P11908 | PRPS2 | 1.301 | 0.001020049 |
| 85 | 50.76 | P46777 | RPL5 | 1.296 | 0.003728950 |
| 86 | 78.08 | P53396 | ACLY | 1.296 | 0.000537925 |
| 87 | 20.62 | P61604 | HSPE1 | 1.294 | 0.020061459 |
| 88 | 18.23 | P25398 | RPS12 | 1.293 | 0.011102054 |
| 89 | 20.05 | P23588 | EIF4B | 1.293 | 0.006337495 |
| 90 | 13.18 | O60869 | EDF1 | 1.292 | 0.000283804 |
| 91 | 21.29 | P30038 | ALDH4A1 | 1.289 | 0.000000002 |
| 92 | 37.09 | P35232 | PHB | 1.288 | 0.000009984 |
| 93 | 26.77 | A0AVT1 | UBA6 | 1.288 | 0.005048157 |
| 94 | 27.40 | P36542 | ATP5F1C | 1.287 | 0.000645354 |
| 95 | 12.85 | O14558 | HSPB6 | 1.285 | 0.042862452 |
| 96 | 8.88 | L0R6Q1 | SLC35A4 | 1.285 | 0.000000145 |
| 97 | 49.19 | O94979 | SEC31A | 1.281 | 0.000003350 |
| 98 | 5.15 | Q13601 | KRR1 | 1.281 | 0.008408696 |
| 99 | 6.62 | Q9BV57 | ADI1 | 1.279 | 0.009962407 |
| 100 | 21.55 | P14735 | IDE | 1.279 | 0.001848224 |
| 101 | 29.59 | Q93008 | USP9X | 1.279 | 0.003269024 |
| 102 | 7.73 | P63173 | RPL38 | 1.278 | 0.000034192 |
| 103 | 15.85 | Q9UIA9 | XPO7 | 1.277 | 0.027553673 |
| 104 | 12.88 | Q16363 | LAMA4 | 1.277 | 0.007724512 |
| 105 | 14.64 | P05090 | APOD | 1.276 | 0.005927724 |
| 106 | 27.29 | P17174 | GOT1 | 1.275 | 0.000283819 |
| 107 | 4.55 | Q9NX14 | NDUFB11 | 1.275 | 0.000120465 |
| 108 | 16.58 | Q9H4A6 | GOLPH3 | 1.274 | 0.000649024 |
| 109 | 23.18 | P13807 | GYS1 | 1.273 | 0.005922166 |
| 110 | 42.31 | Q7Z2W4 | ZC3HAV1 | 1.271 | 0.000128941 |
| 111 | 35.08 | Q96AG4 | LRRC59 | 1.269 | 0.003926993 |
| 112 | 16.28 | P62910 | RPL32 | 1.266 | 0.010644488 |
| 113 | 10.08 | P40616 | ARL1 | 1.266 | 0.000191877 |
| 114 | 3.53 | P48509 | CD151 | 1.266 | 0.019184719 |
| 115 | 11.45 | Q86VB7 | CD163 | 1.266 | 0.000381456 |
| 116 | 209.24 | Q9Y490 | TLN1 | 1.264 | 0.038890005 |
| 117 | 67.32 | P16615 | ATP2A2 | 1.264 | 0.000999939 |
| 118 | 53.93 | P50851 | LRBA | 1.264 | 0.001443571 |
| 119 | 5.22 | Q9NX58 | LYAR | 1.264 | 0.000015705 |
| 120 | 14.07 | Q03519 | TAP2 | 1.263 | 0.033537807 |
| 121 | 16.75 | Q9Y2Q3 | GSTK1 | 1.262 | 0.000514350 |
| 122 | 38.93 | Q16851 | UGP2 | 1.262 | 0.020046777 |
| 123 | 12.53 | Q5SRE5 | NUP188 | 1.261 | 0.018658433 |
| 124 | 37.91 | O94826 | TOMM70 | 1.259 | 0.000409440 |
| 125 | 5.44 | P82930 | MRPS34 | 1.258 | 0.000531688 |
| 126 | 10.67 | Q15042 | RAB3GAP1 | 1.257 | 0.000007589 |
| 127 | 13.86 | Q9UL15 | BAG5 | 1.256 | 0.012252889 |
| 128 | 14.32 | Q6P1M0 | SLC27A4 | 1.256 | 0.000258492 |
| 129 | 24.68 | Q15067 | ACOX1 | 1.255 | 0.000193904 |
| 130 | 9.24 | P83436 | COG7 | 1.255 | 0.010136752 |
| 131 | 16.39 | P25311 | AZGP1 | 1.254 | 0.004002232 |
| 132 | 15.34 | Q9Y6Q5 | AP1M2 | 1.254 | 0.005561117 |
| 133 | 42.76 | Q9HCC0 | MCCC2 | 1.252 | 0.000162292 |
| 134 | 31.71 | P09622 | DLD | 1.251 | 0.001775561 |
| 135 | 20.11 | Q6NVY1 | HIBCH | 1.251 | 0.012507038 |
| 136 | 32.78 | Q14573 | ITPR3 | 1.250 | 0.000141119 |
| 137 | 9.49 | Q9NRN7 | AASDHPPT | 1.249 | 0.001992682 |
| 138 | 5.77 | Q9BZX2 | UCK2 | 1.248 | 0.000026403 |
| 139 | 10.07 | O75381 | PEX14 | 1.247 | 0.007518569 |
| 140 | 5.11 | Q676U5 | ATG16L1 | 1.247 | 0.001878373 |
| 141 | 25.36 | P27635 | RPL10 | 1.246 | 0.000902375 |
| 142 | 26.98 | Q6P996 | PDXDC1 | 1.246 | 0.000947930 |
| 143 | 14.30 | Q6UXG2 | KIAA1324 | 1.246 | 0.003891904 |
| 144 | 6.69 | Q96HD1 | CRELD1 | 1.245 | 0.000022880 |
| 145 | 37.89 | O95831 | AIFM1 | 1.244 | 0.000321410 |
| 146 | 16.83 | Q8N4A0 | GALNT4 | 1.244 | 0.000629802 |
| 147 | 59.70 | P51659 | HSD17B4 | 1.243 | 0.000000161 |
| 148 | 9.25 | Q8WUY3 | PRUNE2 | 1.243 | 0.000000413 |
| 149 | 8.43 | Q05655 | PRKCD | 1.242 | 0.006684120 |
| 150 | 20.10 | P27144 | AK4 | 1.241 | 0.024009165 |
| 151 | 5.17 | Q96E11 | MRRF | 1.241 | 0.000095921 |
| 152 | 13.86 | Q9HAV7 | GRPEL1 | 1.239 | 0.001925287 |
| 153 | 8.41 | Q9BY67 | CADM1 | 1.238 | 0.003295701 |
| 154 | 5.96 | Q9UEW8 | STK39 | 1.238 | 0.000366590 |
| 155 | 9.96 | P60866 | RPS20 | 1.237 | 0.000018331 |
| 156 | 13.96 | Q9BXW7 | HDHD5 | 1.235 | 0.000005291 |
| 157 | 13.76 | Q99848 | EBNA1BP2 | 1.235 | 0.001810876 |
| 158 | 26.71 | P22033 | MMUT | 1.235 | 0.000015268 |
| 159 | 73.06 | P06576 | ATP5F1B | 1.233 | 0.005654449 |
| 160 | 47.19 | Q99959 | PKP2 | 1.231 | 0.010611852 |
| 161 | 51.70 | P47897 | QARS | 1.230 | 0.002816393 |
| 162 | 24.46 | Q969V3 | NCLN | 1.230 | 0.012495596 |
| 163 | 13.16 | Q9UNE7 | STUB1 | 1.228 | 0.000177282 |
| 164 | 2.03 | Q9H871 | RMND5A | 1.228 | 0.001109332 |
| 165 | 9.20 | O96013 | PAK4 | 1.226 | 0.048019786 |
| 166 | 20.49 | P61289 | PSME3 | 1.225 | 0.005866418 |
| 167 | 14.79 | P62851 | RPS25 | 1.225 | 0.024556652 |
| 168 | 36.24 | Q13596 | SNX1 | 1.225 | 0.000301537 |
| 169 | 28.54 | Q10713 | PMPCA | 1.225 | 0.000580031 |
| 170 | 7.68 | Q9BSH5 | HDHD3 | 1.224 | 0.002581596 |
| 171 | 15.45 | P10586 | PTPRF | 1.222 | 0.000000107 |
| 172 | 8.91 | Q14690 | PDCD11 | 1.221 | 0.009128131 |
| 173 | 14.29 | Q96DE0 | NUDT16 | 1.219 | 0.002659163 |
| 174 | 34.85 | Q06210 | GFPT1 | 1.219 | 0.001396951 |
| 175 | 24.86 | O00116 | AGPS | 1.218 | 0.038634229 |
| 176 | 27.88 | P00505 | GOT2 | 1.217 | 0.001952445 |
| 177 | 15.53 | Q92506 | HSD17B8 | 1.215 | 0.000005332 |
| 178 | 12.21 | Q53H96 | PYCR3 | 1.214 | 0.000000088 |
| 179 | 20.81 | P23368 | ME2 | 1.214 | 0.000424127 |
| 180 | 15.43 | P62899 | RPL31 | 1.212 | 0.009387795 |
| 181 | 151.36 | Q00610 | CLTC | 1.211 | 0.018359007 |
| 182 | 17.67 | Q6YN16 | HSDL2 | 1.211 | 0.016105265 |
| 183 | 7.84 | P51571 | SSR4 | 1.211 | 0.007930540 |
| 184 | 11.01 | Q96GQ5 | C16orf58 | 1.211 | 0.000679013 |
| 185 | 6.50 | Q7L5Y1 | ENOSF1 | 1.211 | 0.000045648 |
| 186 | 32.04 | Q16822 | PCK2 | 1.210 | 0.017986755 |
| 187 | 2.30 | A6NDU8 | C5orf51 | 1.209 | 0.000003899 |
| 188 | 5.37 | Q9NX55 | HYPK | 1.207 | 0.000783620 |
| 189 | 5.89 | P60468 | SEC61B | 1.207 | 0.000360065 |
| 190 | 8.40 | P17568 | NDUFB7 | 1.207 | 0.000472443 |
| 191 | 25.70 | P51114 | FXR1 | 1.207 | 0.036923085 |
| 192 | 11.15 | Q16625 | OCLN | 1.207 | 0.000427576 |
| 193 | 17.07 | Q9UDR5 | AASS | 1.206 | 0.002463121 |
| 194 | 6.12 | P12236 | SLC25A6 | 1.205 | 0.004857180 |
| 195 | 20.09 | Q10567 | AP1B1 | 1.203 | 0.000004013 |
| 196 | 48.54 | Q9BSJ8 | ESYT1 | 1.203 | 0.005600097 |
| 197 | 49.77 | P61247 | RPS3A | 1.202 | 0.004162970 |
| 198 | 26.95 | P21589 | NT5E | 1.201 | 0.014832553 |
| 199 | 59.42 | Q92896 | GLG1 | 1.201 | 0.030982175 |
| 200 | 70.16 | Q13423 | NNT | 1.200 | 0.001449643 |
| 201 | 21.75 | Q8NFW8 | CMAS | 1.200 | 0.014375637 |
| 202 | 3.52 | Q92766 | RREB1 | 1.200 | 0.012078166 |
| 203 | 13.99 | Q9BUT1 | BDH2 | 0.833 | 0.000232959 |
| 204 | 78.81 | P48681 | NES | 0.833 | 0.000613953 |
| 205 | 23.20 | P09211 | GSTP1 | 0.832 | 0.000107263 |
| 206 | 49.73 | Q5TZA2 | CROCC | 0.832 | 0.000000141 |
| 207 | 12.28 | O94901 | SUN1 | 0.832 | 0.000000133 |
| 208 | 62.90 | Q13263 | TRIM28 | 0.828 | 0.000139692 |
| 209 | 13.03 | P80404 | ABAT | 0.827 | 0.000056197 |
| 210 | 33.99 | P38919 | EIF4A3 | 0.826 | 0.000000679 |
| 211 | 25.33 | P55268 | LAMB2 | 0.826 | 0.000000001 |
| 212 | 8.77 | O95777 | LSM8 | 0.824 | 0.000000000 |
| 213 | 8.92 | P01742 | IGHV1-69 | 0.823 | 0.000079188 |
| 214 | 6.03 | P31323 | PRKAR2B | 0.823 | 0.000269037 |
| 215 | 28.92 | Q16658 | FSCN1 | 0.822 | 0.000002247 |
| 216 | 5.10 | Q8IZP9 | ADGRG2 | 0.820 | 0.000000846 |
| 217 | 54.32 | P78347 | GTF2I | 0.819 | 0.000002011 |
| 218 | 16.49 | Q15417 | CNN3 | 0.818 | 0.000026426 |
| 219 | 24.34 | P62140 | PPP1CB | 0.816 | 0.000000003 |
| 220 | 22.18 | Q9NTI5 | PDS5B | 0.815 | 0.000057497 |
| 221 | 5.41 | Q15257 | PTPA | 0.812 | 0.000148747 |
| 222 | 3.76 | P62312 | LSM6 | 0.807 | 0.000000002 |
| 223 | 82.04 | P35580 | MYH10 | 0.804 | 0.000001784 |
| 224 | 25.97 | P0DP25 | CALM3 | 0.803 | 0.000008056 |
| 225 | 45.67 | P46821 | MAP1B | 0.800 | 0.000004721 |
| 226 | 16.29 | Q9Y696 | CLIC4 | 0.795 | 0.000048374 |
| 227 | 20.68 | Q93052 | LPP | 0.793 | 0.000020472 |
| 228 | 42.26 | Q02952 | AKAP12 | 0.793 | 0.000021226 |
| 229 | 49.75 | Q9Y3I0 | RTCB | 0.791 | 0.000006799 |
| 230 | 38.79 | Q9Y4G6 | TLN2 | 0.791 | 0.000000199 |
| 231 | 16.89 | Q9Y2T3 | GDA | 0.788 | 0.000011874 |
| 232 | 19.18 | O75781 | PALM | 0.786 | 0.000002657 |
| 233 | 8.77 | P07108 | DBI | 0.784 | 0.000000002 |
| 234 | 19.20 | P12821 | ACE | 0.783 | 0.000000095 |
| 235 | 108.89 | P20700 | LMNB1 | 0.779 | 0.000000034 |
| 236 | 18.45 | P27348 | YWHAQ | 0.776 | 0.000005735 |
| 237 | 36.42 | P36871 | PGM1 | 0.775 | 0.000000000 |
| 238 | 7.75 | O00592 | PODXL | 0.775 | 0.000376937 |
| 239 | 7.15 | P40938 | RFC3 | 0.771 | 0.000000079 |
| 240 | 21.64 | Q14194 | CRMP1 | 0.769 | 0.000023813 |
| 241 | 24.71 | O43175 | PHGDH | 0.769 | 0.000078541 |
| 242 | 11.20 | O95810 | CAVIN2 | 0.766 | 0.000000012 |
| 243 | 52.68 | Q92499 | DDX1 | 0.761 | 0.000002731 |
| 244 | 4.02 | Q14956 | GPNMB | 0.761 | 0.000000003 |
| 245 | 34.15 | Q16401 | PSMD5 | 0.758 | 0.000004030 |
| 246 | 16.14 | H7BZ55 | CROCC2 | 0.755 | 0.000000788 |
| 247 | 10.38 | P50897 | PPT1 | 0.754 | 0.000000014 |
| 248 | 27.47 | Q6NZI2 | CAVIN1 | 0.747 | 0.000000070 |
| 249 | 73.14 | P09874 | PARP1 | 0.745 | 0.000000008 |
| 250 | 8.24 | P22413 | ENPP1 | 0.744 | 0.000000548 |
| 251 | 11.98 | Q03135 | CAV1 | 0.736 | 0.000000019 |
| 252 | 9.45 | P42773 | CDKN2C | 0.730 | 0.000000804 |
| 253 | 47.32 | P08473 | MME | 0.726 | 0.000003429 |
| 254 | 17.33 | P32456 | GBP2 | 0.724 | 0.000000344 |
| 255 | 14.58 | P23219 | PTGS1 | 0.721 | 0.000009435 |
| 256 | 22.76 | Q9NZN4 | EHD2 | 0.717 | 0.000000125 |
| 257 | 27.57 | P27695 | APEX1 | 0.704 | 0.000000040 |
| 258 | 14.47 | P21266 | GSTM3 | 0.682 | 0.000000467 |
| 259 | 4.57 | P09936 | UCHL1 | 0.669 | 0.000000095 |
| 260 | 25.76 | P04275 | VWF | 0.660 | 0.000000000 |
| 261 | 15.00 | Q99584 | S100A13 | 0.639 | 0.000000000 |
| 262 | 13.76 | Q9Y6M1 | IGF2BP2 | 0.637 | 0.000000034 |
| 263 | 21.15 | Q9BW30 | TPPP3 | 0.615 | 0.000000000 |
